# The Activity Concentrations and Radium Equivalent Activity in Soil Samples Collected from the Eastern Part of Basrah Governorate in Southern Iraq

**DOI:** 10.1155/2018/2541020

**Published:** 2018-05-28

**Authors:** Rasha S. Ahmed, Raghad S. Mohammed, Rana O. Abdaljalil

**Affiliations:** ^1^Department of Physiology and Medical Physics, College of Medicine, Al-Nahrain University, P. O. Box 70010, Alkadhimiya, Baghdad, Iraq; ^2^Department of Physics, College of Science, Al-Mustansiriyah University, Baghdad, Iraq

## Abstract

Clay soil samples (0, 30, and 60 cm depths) were collected from two districts (Abu Al Khasib and Ad Dayr) in Basrah governorate in southern Iraq for gamma-ray spectroscopy. The activity concentrations for natural existing radionuclides in 18 soil samples were measured using high-purity germanium detector HPGe. From the obtained results of *γ*-ray spectroscopy, the ^238^U activity concentrations were found to be ranging from 2.4 to 5.6 ppm with an average of 3.5 ppm in Abu Al Khasib and ranging from 2.1 to 4.5 ppm with an average of 2.9 ppm in Ad Dayr. ^232^Th concentrations were ranging from 3.6 to 7.5 ppm with an average of 4.7 ppm in Abu Al Khasib and ranging from 3.7 to 7.9 ppm with an average of 5.0 ppm in Ad Dayr. ^40^K concentration was ranging from 0.1% to 2.0% with an average of 1.2% in Abu Al Khasib and ranging from 0.9% to 1.8% with an average of 1.3% in Ad Dayr. High ^238^U and ^226^Ra concentration levels were recorded in both study regions. The concentrations of ^232^Th are within the normal limits in both regions. High levels of ^40^K were recorded in some locations. Generally, in most locations, ^40^K activity was within normal ranges. The radium equivalent activity, the external hazard index, the internal hazard index, and the radioactivity level index were calculated to estimate the radiation hazard in Basrah. The estimated radiation hazard indices were within normal limits, except the radioactivity level index, which shows elevated values. The obtained results were compared with other countries and with the worldwide median certified values.

## 1. Introduction

Human beings are exposed to natural radiation sources. The natural sources include the cosmic radiations that come from the sun that start as charged particles, which interact with the earth's atmosphere to produce gamma and beta radiations. Natural radioactive materials are also included in soil, water, and vegetation known as terrestrial radiation. In addition to the cosmic and terrestrial sources, humans also have radioactive isotopes inside their bodies since childbirth. Humans are also exposed to the man-made radiation used in medical processes such as radiation treatment, nuclear medicine, and diagnostic X-rays. Besides medical applications, man-made radiations are received from nuclear-power reactors and facilities involved in nuclear weapons. The major risky source is the usage of depleted uranium in weapons used in wars, which constitutes a great danger for the population. DU has a half-life of 4.4 billion years and is used in nuclear reactors and in the manufacture of nuclear weapons.

A number of factors affect the terrestrial radiation exposure such as the concentration of radionuclides in soil. The most essential primal radionuclides are ^238^U with half-life of 4.5 × 10^9^years, ^232^Th with half-life 1.405 × 10^10^years, and ^40^K with half-life of 1.277 ×10^9^ years [1]. The concentration of radionuclides in air, water, soil, and rock were discussed in several studies [2–5].

Basrah, a city in southern Iraq of 1.6 million and 19.070 km^2^, has suffered from the spread of cancerous injuries and birth defects. High number of cancer cases among whom are children who are more sensitive to radiation than adults were recorded. A complete of 8,748 occasions were accumulated, 72.9% being residents of Basrah governorate, and 27.1% from an exterior. The documented numbers in the years 2005, 2006, 2007, and 2008 were 1,850, 2,155, 2,410, and 2,333 cases, respectively, males and females accounting for 48.1% and 51.9% of cases [6].

Abu Al Khasib (30.252 ^0^N, 48.0737 ^0^E, 1152 Km^2^) and Ad Dayr (30.829 ^0^N, 47.574 ^0^E, 825 Km^2^) are considered as an important agricultural districts in Basrah. Additionally, Ad Dayer is deemed to be a significant archaeological and religious region and visited by many tourists each day. Formerly, we use the same samples that we will adopt in this work [7] to estimate the activity concentrations in Bq/kg; the average outdoor external dose, average indoor external dose, total average value of the external dose, average annual outdoor effective dose, average annual indoor effective dose, and averaged total annual effective dose have been estimated. Values for total average value of the external dose and averaged total annual effective dose were higher than the worldwide median in both regions. Additionally, the excess lifetime cancer risk has been also assessed and found to be higher than the worldwide averages.

As both areas are important and considered as a source to equip the Iraqi provinces with agricultural crops, this work has been adopted to extend the results obtained in previously published work [7], to estimate additional radiation hazard indices that may affect the human health. In the present study, we aim to estimate the activity concentrations for radionuclides in ppm in clay soil samples for ^238^U, ^226^Ra, ^232^Th, and ^40^K and compared the results with the international confirmed surveys. Moreover, to distinguish regions that might be radiologically unsafe, the radium equivalent activity, the external hazard index, the internal hazard index, and the radioactivity level index were estimated and compared with each other and with worldwide estimated values.

## 2. Materials and Methods

### 2.1. Sample Collection and Preparation

**Figure 1:**
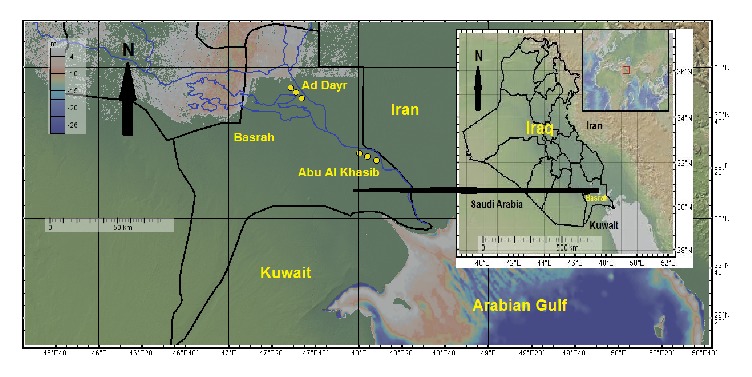
Map of the surveyed area conducted using geomapapp [7].

A total of 18 (9 from Abu Al Khasib and 9 from Ad Dayr) clay soil samples were collected from the study regions as shown in Figure 1 [7]. As shown in Figure 1, only three sites were indicated in each of the sampling regions, since, from each indicated site, three samples were collected from (0, 30, 60 cm) depths using a plastic hand trowel. In each study region, a 1 m^2^ sampling site was covered, each site located 1 m straight forward from the other. Samples were positioned in coded black plastic case about 1 kg each. The code's explanations are listed in Table 1. Samples were air-dried up on a paten for five days until every wetness was dissolved. The moisture-less samples then ground to soften fine powder employing a grinder and sifted through a (2mm) mesh and stored for one month to permit total growth of ^226^Ra with its daughters ^222^Rn, ^214^Pb, and ^214^Bi before sending to Gamma Laboratory for the Ministry of Environment Radiation Protection Center (RPC) in Iraq, Baghdad.

### 2.2. Measurement by Gamma-Ray Spectrometry

In Gamma Laboratory, the activity concentrations for samples were measured using Canberra high-purity germanium (HPGe) detector, including mixed gamma source. The mixed gamma-ray source contains ^241^Am, ^109^Cd, ^57^Co, ^57^Co, ^139^Ce, ^113^Sn, ^137^Cs, ^88^Y, ^60^Co, ^60^Co, ^85^Sr radionuclides with their energies and activities listed in Table 2.

**Figure 2:**
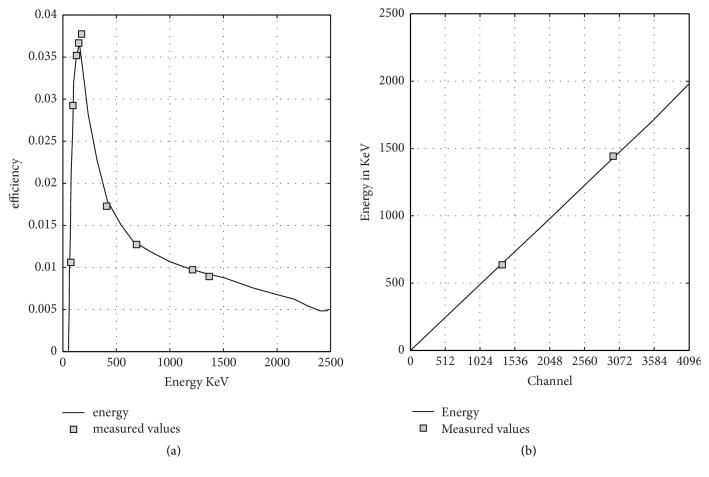
(a) The linear scale of the efficiency calibration curve for HPGe detector (order of the polynomial is 4 with *R*^2^ = 0.709). (b) The energy calibration curve for HPGe detector.

As it is arduous to split the recorded low activity samples signal from the background radiation, the calculations were carried out employing a Pb shielding with a settled bottom and portable cover. The results for all samples were estimated using the efficiency as a function of energy, the energy as a function of number of channels, and the full width half maxima FWHM as a function of number of channels calibrations. The efficiency calibration was performed using different energy peaks to cover the energy range from 60 up to 2500 keV (Figure 2(a)). The efficiency *ϵ* of the counting for energies *E* utilized in the calculations is given by

(1) ln⁡ϵ=−1.943×102+1.264×102ln⁡E−3.089×101ln⁡E2+3.309ln⁡E3−1.319×10−1ln⁡E4

The energy calibration was established with 512, 1024, 1536, 2048, 2560, 3072, 3584, and 4096 channel numbers (Figure 2(b)) and well fitted with the first degree function as

(2) $$\begin{array}{c} Energy=3.056\times 10^{-1}+5.001\times 10^{-1}\times Ch\;keV \end{array}$$

Finally, the FWHM calibration performed uses the following expression:

(3) $$\begin{array}{c} FWHM=6.013\times 10^{-1}+3.517\times 10^{-2}E^{1/2}\;keV \end{array}$$

The calibrations are saved and used in the acquisition program to apply the corrections for each sample.

**Figure 3:**
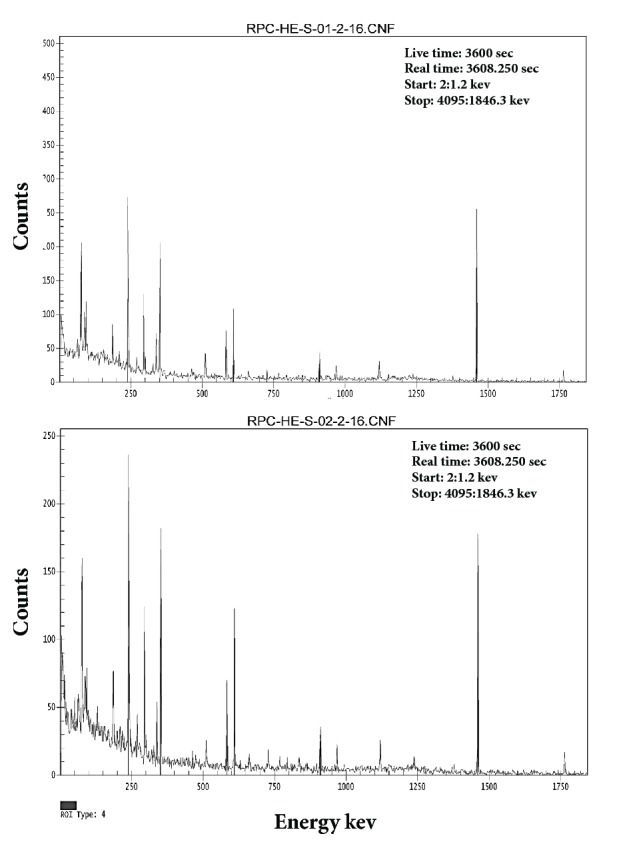
The number of counts as a function of energy of the radionuclides in samples 1 and 2, obtained by HPGe detector, and analyzed through Genie 2k Canberra software.

**Figure 4:**
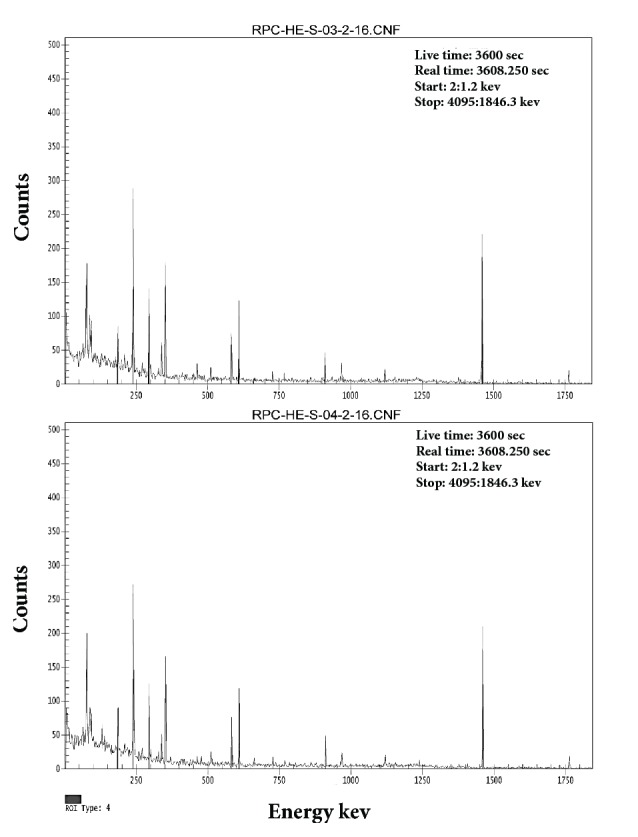
Same as Figure 3 for samples 3 and 4.

**Figure 5:**
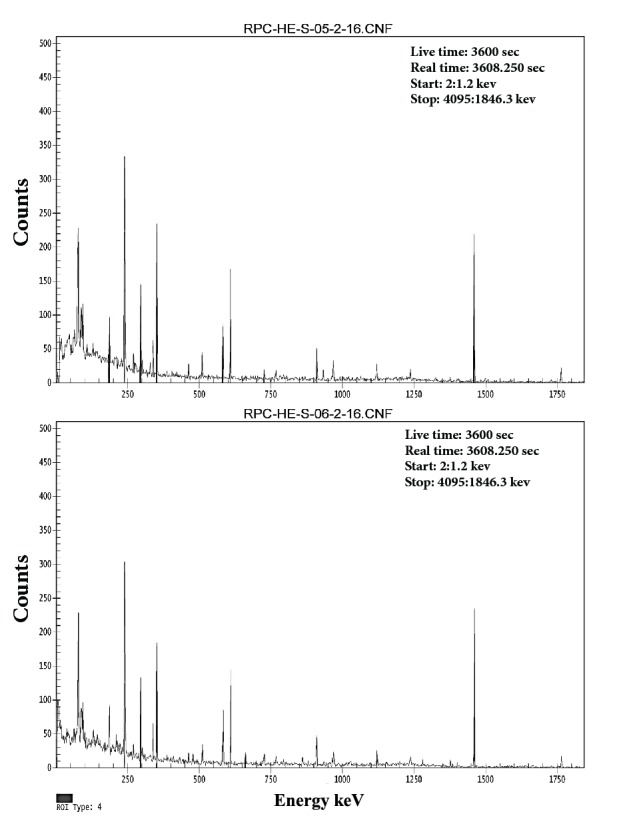
Same as Figure 3 for samples 5 and 6.

The average activity concentration of ^238^U was measured from the median peak energies of 351.9 keV of ^214^Pb and 186.2 keV of ^226^Ra. Correspondingly, the average activity concentration of ^232^Th was estimated from the median peak energies of 238 keV of ^212^Pb, 727.2 keV of ^212^Bi, and 911.6 keV of ^228^Ac [23]. All other radionuclides concentrations were estimated directly from its known peaks, as seen in Figures 3–5 for 1, 2, 3, 4,5,6 sample numbers. The calculated gamma spectra have been analyzed using Genie 2k Canberra software.

## 3. Results and Discussions

### 3.1. The Activity in Clay Soil from Abu Al Khasib and Ad Dayr

18 clay soil samples were collected from the indicated locations in Figure 1 using Global Positioning System (GPS). Three samples were collected from each indicated site at 0, 30, and 60 cm depths. The measured activity concentrations of ^238^U, ^232^Th, ^40^K in the clay soil samples were listed in Table 3. The levels of general radioactivity due to isotopes were listed in Bq/kg [7]. The concentrations of specific radioisotope in the samples were also measured in parts per million (ppm). Additionally, the maximum (max.), minimum (min.), average (ave.), standard deviation (stdev.), median (med.), and median absolute deviation (med. abs. div.) for the activity concentrations in Bg/kg and ppm have been included.

The concentrations in ppm of ^238^U, ^232^Th, ^40^K were calculated from the measured activities in Bq/kg (Table 3) using the following experimental formula [24]:

(4) $$\begin{array}{c} A\left(\;ppm\;\right)=C\left(\;\frac{Bq}{kg}\;\right)\lambda =C\left[\;\left(\;\frac{M_{w}}{N\ln\left(2\right)}\;\right)t_{1/2}\;\right]\times 10^{6} \end{array}$$

where C is the activity concentration in Bq/kg, *M*_*w*_ is the molecular weight (g/mol), *N* is Avogadro's number, and *t*_1/2_ is the half-life in seconds. The measured conversion factors *λ* for each radionuclide has been listed in Table 4, in addition to half-lives, and atomic masses used in the calculations.

Abu Al Khasib had 240,300 inhabitants and Ad Dayr 130,000 citizens in 2012. Statistics recorded a rise at the rate of birth defects from 3.2 cases per 1,000 births in 1990 to 22 cases per 1,000 births in 2000. Basrah witnessed the birth of 300 deformed children within one year [6]. In both Abu Al Khasib and Ad Dayr, the activity concentration has been measured and analyzed to focus on the radiological environment of both Abu Al Khasib and Ad Dayr.

Uranium can be found spontaneously in the environment in very little magnitudes in air, soil, rocks, and water. In air, the uranium concentrations are very low. In water, most of the uranium is thawed uranium that is obtained from rocks and soil that the water moves over. The quantities of uranium in drinking water are usually low. Uranium is found in soils in changing concentrations that are typically very low. People constantly encounter exposure to a particular magnitude of uranium from food, air, soil, and water, as it spontaneously existed in all these elements. Individuals who settle nearby hazardous waste locations and eat yields farmed in contaminated soil may suffer a stronger exposure than other people. When humans are exposed to uranium radionuclides that are created throughout radioactive decay for a long interval, they possibly will develop cancer. The possibilities of obtaining cancer are much higher when persons are exposed to enriched uranium, since that is a further radioactive form of uranium. This form of uranium provides harmful radiation, which can cause people to evolve cancer within a few years.

As shown in Table 3, ^238^U activity concentration is ranging from 29.50 (2.4 ppm) to 69.00 Bq/kg (5.6 ppm) with an average of 43.56 Bq/kg (3.5 ppm) in Abu Al Khasib and ranging from 26.50 (2.1 ppm) to 56.10 Bq/kg (4.5 ppm) with an average of 35.53 Bq/kg (2.9 ppm) in Ad Dayr. ^232^Th concentration is ranging from 14.70 (3.6 ppm) to 30.80 Bq/kg (7.5 ppm) with an average of 19.39 Bq/kg (4.7 ppm) in Abu Al Khasib and ranging from 15.30 (3.7 ppm) to 32.20 Bq/kg (7.9 ppm) with an average of 20.33 Bq/kg (5.0 ppm) in Ad Dayr. ^40^K concentration is ranging from 25.80 (0.1%) to 528.20 Bq/kg (2.0%) with an average of 321.76 Bq/kg (1.2%) in Abu Al Khasib and ranging from 241.80 (0.9%) to 461.40 Bq/kg (1.8%) with an average of 337.02 Bq/kg (1.3%) in Ad Dayr. The results of the activity concentrations in Bq/kg (Table 3) were compared with estimated values in the soil of different countries (Table 5). Furthermore, the worldwide average data were also included for comparison. High ^238^U concentration levels were recorded in both study regions, greater than the world average and greater than some countries listed in the table. The concentrations of ^232^Th, ^40^K are within the normal limits in both regions. In some locations (sites 1 and 6) high levels of ^40^K concentrations were recorded.

*Ra* exists at absolute low levels in the natural environ, substantially, in all rocks, soil, and water; furthermore, it is a decay product of uranium and thorium. Hence, where uranium and thorium appear at fairly elevated grades in rocks or soil, radium is as well found at elevated levels. As shown in Table 5, ^226^Ra activity ranging from 37.40 to 99.60 Bq/kg with an average of 58.44 Bq/kg in Abu Al Khasib and ranging from 32.00 to 75.40 Bq/kg with an average of 45.71 Bq/kg in Ad Dayr. According to UNSCEAR report [10], extreme high levels of radium were recorded in both regions, higher than the world average and some of the countries included. Back to Figures 3, 4, and 5, cesium activities of 4.60 and 1.80 Bq/kg were recorded in Abu Al Khasib (samples 3 and 6) and an activity of 1.10 Bq/kg in Ad Dayr (sample 10).

In Table 6, the present study massic concentrations in clay soil of ^238^U, ^232^Th, and ^40^K were compared with randomly selected countries evaluations in clay soil around the world and with the worldwide averaged values. The averaged ^238^U (ppm) concentration attained in this study is greater than the world average value and also greater than some listed countries. The concentration of ^232^Th (ppm) is lower than the world average but higher than some listed countries. The K(%) concentration is lower than the world average and lower than the listed countries. Both of the study locations are farming areas in which plowing and mixing of the soil take place to prepare it for agriculture; as a result, there is no particular order of contamination with depth.

### 3.2. The Radiation Hazard Indices

It is significant to evaluate the *γ*-ray radiation hazards of soil to human beings on Abu Al Khasib and Ad Dayr. The *γ*-ray radiation hazards due to the natural radionuclides ^226^Ra, ^232^Th, and ^40^K were assessed by various radiation hazard indices. Radium equivalent activity *Ra*_*eq*_ is widely used to identify the consistency of radiation exposure; consequently, any radium equivalent activity concentration that passes 370 Bq/Kg may raise radiation hazards. In the present study, *Ra*_*eq*_ was determined in Bq/kg using the following expression [25]:

(5) $$\begin{array}{c} {Ra}_{eq}\left(\;Bq{kg}^{-1}\;\right)=0.077C_{K}+C_{Ra}+1.43C_{Th} \end{array}$$

where *C*_*K*_, *C*_*Ra*_, and *C*_*Th*_ are the activities for potassium, radium, and thorium, respectively.

The Ra activity concentration and *Ra*_*eq*_ in (*Bq*/*Kg*) calculated for the samples under study are listed in Table 7. The recorded values are ranging from 60.46 to 184.32 (*Bq*/*Kg*) with an average 110.94 (*Bq*/*Kg*) in Abu Alkhasib and from 77.24 to 156.97 (*Bq*/*Kg*) with an average 100.74 (*Bq*/*Kg*) in Ad Dayer. These values are less than the approved maximum value in building materials of 350 (*Bq*/*Kg*) [26]. Further important criterion used to estimate the level of gamma-ray radiation is defined by the external hazard index (H_ex_) and the internal hazard index (H_in_) which is defined as follows [26–28]:

(6) Hex=CU370 Bqkg−1+CTh259 Bqkg−1+CK4810 Bqkg−1

(7) Hin=CU185 Bqkg−1+CTh259 Bqkg−1+CK4810 Bqkg−1

H_ex_ is used to calculate the indoor radiation dose value due to the outward exposure to *γ*-radiation released by the natural radionuclides in the formation building substances of the lodging places. Moreover, the internal exposure to ^222^ Rn and its radioactive progeny are managed by *H*_*in*_. The radiation hazard considered as insignificant if both H_ex_ and H_in_ are less than one [29, 30]; this is very significant to keep the concentration levels of radon and its short-lived daughters low enough for the respiratory organs of humans living comparable to the recorded international levels of 40 Bq m^−3^ [10]. For both H_ex_ and H_in_ to be less than one, the maximum values of Ra_eq_ must be less than 370 Bq/kg and 185 Bq/kg, respectively. The estimated results of H_ex_ for clay soil samples are ranging from 0.5 to 0.16 with an average value of 0.3 (Bq/kg) in Abu Al Khasib and ranging from 0.21 to 0.42 with an average value of 0.27 (Bq/kg) in Ad Dayer. Also, H_in_ ranges from 0.26 to 0.77 with an average value of 0.46 (Bq/kg) in Abu Al Khasib and ranges from 0.29 to 0.63 with an average value of 0.40 (Bq/kg) in Ad Dayer (Table 7). This implies that the external and internal hazard indices estimated in this study are below the recommended limit.

The radioactivity level index *I*_*γ*_ is used to monitor radiation inside the human body and to compute the risky level of radionuclides in the human body when exposed to an amount of indoor or outdoor annual effective doses of *γ*-radiations from radioactive nuclides in soils. The estimated values of *I*_*γ*_ should be less than or equal to one to make sure the soil environment is hazard-free. In this work, *I*_*γ*_ calculated to certify that such radiation does not exceed the world high dose values [31]. Values of *I*_*γ*_ were calculated according to the following formula [32]:

(8) $$\begin{array}{c} I_{\gamma }=\frac{C_{U}}{150\;Bq{kg}^{-1}}+\frac{C_{Th}}{100\;Bq{kg}^{-1}}+\frac{C_{K}}{1500\;Bq{kg}^{-1}} \end{array}$$

As illustrated in Table 7  *I*_*γ*_ is ranging from 0.41 to 1.32 with an average of 0.8 in Abu Al Khasib and ranging from 0.57 to 1.13 with an average of 0.73 in Ad Dayer. In most soil samples, the calculated values of *I*_*γ*_ are close to or higher than 1, which implies that these soil samples should be avoided and not used in agriculture or in building materials [31].

The results show that the increased incidence of cancer and neonatal abnormalities is related to the higher concentration of radionuclides in the soil, as the rates of cancer and deformities were not so high before the wars and the presence of military barracks in those areas until the time that the samples have been collected.

## 4. Conclusions 

Estimations on clay soil samples were carried elementally for ^238^U, ^232^Th, ^226^Ra, and ^40^K. 18 samples were collected in a previous work from Abu Al Khasib and Ad Dayr districts in Basrah, Iraq, from different depths (0, 30, and 60 cm). The consequences attained indicate obviously that the mean activities of ^238^U and ^226^Ra radionuclides examined in the soil are high as compared to median levels documented worldwide. ^232^Th, ^40^K activities are low compared to the world's average levels. In addition to that, the achieved evaluations of the radiation hazard indices due to the activity concentrations of ^238^U, ^232^Th, ^226^Ra, and ^40^K indicated that the indices are within normal limits, except the radioactivity level index *I*_*γ*_, which shows elevated values in most samples. Abu Al Khasib and Ad Dayr can be regarded as areas with elevated back radioactive ground radiation.

## Figures and Tables

**Table 1:** Samples regions, depths, and codes indices [7].

Abu Al Khasib									

Samples number	1	2	3	4	5	6	7	8	9
Samples codes	A-S-1	A-30-2	A-60-3	A-S-4	A-30-5	A-60-6	A-S-7	A-30-8	A-60-9
Depth of samples (cm)	0	30	60	0	30	60	0	30	60

Ad Dayer									

Samples number	10	11	12	13	14	15	16	17	18
Samples codes	D-S-1	D-30-2	D-60-3	D-S-4	D-30-5	D-60-6	D-S-7	D-30-8	D-60-9
Depth of samples (cm)	0	30	60	0	30	60	0	30	60

**Table 2:** Mixed gamma source radionuclides used in HPGe detector and its standard properties.

Radionuclide	^241^Am	^109^Cd	^57^Co	^57^Co	^139^Ce	^113^Sn	^137^Cs	^88^Y	^60^Co	^60^Co	^85^Sr
Energy keV	60	88	122.5	136.9	165.7	392	661.6	898	1173.7	1132.8	513.9
Activity kBq	1.605	0.311	0.240	0.030	0.066	0.145	2.019	0.220	2.272	2.272	0.038

**Table 3:** ^238^U, ^232^Th, ^40^K activities in Bq/kg [7] and the estimated values in ppm using *γ*-ray spectroscopy.

Activity concentrations						
Samples codes	^238^U		^232^Th		^40^K	
	Bq/kg	ppm	Bq/kg	ppm	Bq/kg	ppm
1	58.6	4.7	26.9	6.6	522.1	2.0
2	57.3	4.6	18.9	4.6	408.1	1.6
3	40	3.2	18.5	4.5	296.6	1.1
4	31.7	2.6	15.4	3.8	298.4	1.2
5	38.3	3.1	17.2	4.2	240	0.9
6	69	5.6	30.8	7.5	528.2	2.0
7	31.3	2.5	16.8	4.1	303.4	1.2
8	36.3	2.9	15.2	3.7	273.2	1.1
9	29.5	2.4	14.7	3.6	25.8	0.1
max.	69.0	5.6	30.8	7.5	528.2	2.0
min.	29.5	2.4	14.7	3.6	25.8	0.1
ave.	43.6	3.5	19.4	4.7	321.8	1.2
stdev.	20.6	1.6	8.6	2.1	350.9	1.3
med.	38.3	3.1	17.2	4.2	298.4	1.2
med. abs. div.	7	0.6	1.8	0.4	58.4	0.3
10	32.5	2.6	21.4	5.2	341.8	1.3
11	27	2.2	17.3	4.2	300	1.2
12	34.2	2.8	18.3	4.5	322	1.2
13	28.9	2.3	17.3	4.2	241.8	0.9
14	56.1	4.5	32.2	7.9	461.4	1.8
15	30.6	2.5	15.3	3.7	321.4	1.2
16	32	2.6	20.8	5.1	309.8	1.2
17	52	4.2	24.1	5.9	450.2	1.7
18	26.5	2.1	16.3	4.0	284.8	1.1
max.	56.1	4.5	32.2	7.9	461.4	1.8
min.	26.5	2.1	15.3	3.7	241.8	0.9
ave.	35.5	2.9	20.3	5.0	337.0	1.3
stdev.	4.2	0.4	3.6	0.8	40.3	0.1
med.	32	2.6	18.3	4.5	321.4	1.2
med. abs. div.	3.1	0.3	2.5	0.6	21.4	0.1

**Table 4:** The half-lives, atomic masses, and the calculated radioactivity conversion factors from Bq/kg to ppm for  ^238^U, ^232^Th, ^40^K radionuclides.

radionuclide	Half-life (years)	Atomic mass (u)	*λ*
^238^ *U*	4.5 × 10^9^	238.05	0.080901
^232^ *Th*	1.405 × 10^10^	232.03	0.246225
^40^ *K*	1.277 × 10^9^	39.96	3.8585 × 10^−3^

**Table 5:** The average activity concentrations of ^238^U, ^232^Th, ^226^Ra, and ^40^K radionuclides in Bq/kg evaluated by R. S. Mohammed and R. S. Ahmed [7], compared with different countries and worldwide average.

Country	The average activity concentrations (Bq/kg)				
	^238^U	^232^Th	^226^Ra	^40^K	reference
Abu Al Khasib	44	19	58	322	[7]
Ad Dayr	36	20	46	337	[7]
Italy	–	39	323	1,046	[8]
Turkey	29	18	–	580	[9]
Spain	33	49	45	650	[8]
Japan	29	28	68	310	[10]
Belgium	–	28	28	390	[8]
Mexico	23	19	–	530	[11]
India	26	51	–	5.6	[12]
Oman	30	16	–	225	[13]
Algeria	–	–	80	408	[8]
France	37	38	38	599	[8]
Taiwan	18	28	–	479	[8]
Jordan	49	27	–	291	[4]
Egypt	37	18	–	320	[10]
Qatar	23	4.5	–	127	[14]
Thailand	35	30	–	400	[15]
USA	35	35	–	370	[10]
The Netherlands	–	50	–	495	[8]
World	35	30	35	400	[10]

**Table 6:** A comparison of the mass concentrations of ^238^U, ^232^Th, and ^40^K in clay soil samples evaluated in the present study with other locations around the world.

Location	^238^U (ppm)	^232^Th (ppm)	^40^K (%)	references
Present study (Abu Al Khasib)	2.4-5.6	3.6-7.5	0.1-2.0	
Present study (Ad Dayr)	2.1-4.5	3.7-7.9	0.9-1.8	
Cyprus	0.08-7.2	0.25-13.1	0.04-2.91	[16]
Italy	0.16-5.6	0.25-16.8	0.03-5.14	[17]
Bulgaria	0.64-15.2	1.73-39.5	0.05-3.75	[18]
Canada (Rock)	0.8-16.4	1.1-41.0	1.0-6.2	[19]
Jordan	1.76-8.32	5.18-25.4	0.45-1.96	[20]
USA	0.32-11.2	0.98-32.1	0.32-2.28	[18]
Egypt	0.48-9.6	0.49-23.7	0.09-2.12	[18]
Serbia and Montenegro	1.2-6.24	4.45-21.0	0.88-2.99	[21]
Albania	0.48-7.68	0.98-39.5	0.05-3.75	[18]
Australia	1.6-3.8	6-19	0.7-1.9	[22]
World Average	2.64	11.1	1.37	[21]

**Table 7:** The measured values for *Ra*_*eq*_ [7], H_ext_, H_in_ hazard indices in (*Bq*/*Kg*) and the radioactivity level index *I*_*γ*_ for 18 clay soil samples, in addition to its max., min., ave., stdev., med., and med. abs. div. values.

Samples number	^226^ *Ra* (*Bq*/*Kg*)	*Ra*_*eq*_ (*Bq*/*Kg*)	*H*_*ext*_ (*Bq*/*Kg*)	*H*_*in*_ (*Bq*/*Kg*)	*I*_*γ*_
1	76.8	155.50	0.42	0.63	1.13
2	78.4	136.90	0.37	0.58	0.98
3	58	107.29	0.29	0.45	0.77
4	42.8	87.80	0.24	0.35	0.64
5	44.8	87.88	0.24	0.36	0.63
6	99.6	184.32	0.50	0.77	1.32
7	37.4	84.79	0.23	0.33	0.62
8	50.8	93.57	0.25	0.39	0.67
9	37.4	60.41	0.16	0.26	0.41
max.	99.60	184.32	0.50	0.77	1.32
min.	37.40	60.46	0.16	0.26	0.41
ave.	58.44	110.94	0.30	0.46	0.80
stdev.	27.86	67.24	0.18	0.26	0.51
med.	50.8	93.57	0.25	0.39	0.67
med. abs. div.	13.4	13.72	0.04	0.06	0.1
10	39.2	96.12	0.26	0.37	0.70
11	33.8	81.64	0.22	0.31	0.60
12	48.4	99.36	0.27	0.40	0.72
13	36.6	79.96	0.22	0.32	0.58
14	75.4	156.97	0.42	0.63	1.13
15	41.8	88.43	0.24	0.35	0.65
16	41.6	95.20	0.26	0.37	0.69
17	62.6	131.73	0.36	0.53	0.96
18	32	77.24	0.21	0.29	0.57
max.	75.40	156.97	0.42	0.63	1.13
min.	32.00	77.24	0.21	0.29	0.57
ave.	45.71	100.74	0.27	0.40	0.73
stdev.	5.09	13.35	0.04	0.06	0.09
med.	41.6	95.2	0.26	0.37	0.69
med. abs. div.	6.8	13.56	0.04	0.05	0.09

## Data Availability

The data used to support the findings of this study are available from the corresponding author upon request.
